# Family Courts in England & Wales: A population-level evidence base about court users who experience domestic abuse

**DOI:** 10.23889/ijpds.v9i5.2836

**Published:** 2024-09-18

**Authors:** Ludivine Garside

**Affiliations:** 1 University of Bristol Law School

## Objectives

The caseload of family courts in England and Wales includes a growing share of domestic abuse cases. To increase knowledge about such cases, the research uses new administrative datasets and linkages. Trends between 2011-2021 are examined in the context of policy and operational changes.

## Method

The complete set of family court cases is held in HM Courts & Tribunals Services (HMCTS) systems. Some family court data are collected by the Children and Families Court Advisory Support Service (Cafcass), insofar as courts refer the case to Cafcass. Using their new linkage, we conduct a descriptive analysis of domestic abuse cases in England and Wales over the ten years from 2011. This project is one of the first to gain approval from HMCTS to use their de-identified family court data.

## Results

The data linkage allows to describe how court users approach domestic abuse matters, drawing distinctions between: emergency cases; lack of legal representation in court (litigants in person); repeat applications; presence of children in the household. Furthermore, the data show how courts respond through case management, timeliness of decisions and legal outcomes made. The policy context reveals how court users adapted to changes in eligibility for civil legal aid and to changes over how courts operated under lockdown measures.

## Conclusion

Data linkage research is providing an evidence base at population level about court users in England and Wales who experience domestic abuse. This can help inform practice to support families, and policies to ease overburdened courts and avoid returns to court.

